# The function of the inner nuclear envelope protein SUN1 in mRNA export is regulated by phosphorylation

**DOI:** 10.1038/s41598-017-08837-7

**Published:** 2017-08-22

**Authors:** Ping Li, Maria Stumpf, Rolf Müller, Ludwig Eichinger, Gernot Glöckner, Angelika A. Noegel

**Affiliations:** 1 Institute of Biochemistry I, Medical Faculty, University Hospital Cologne, Center for Molecular Medicine Cologne (CMMC) and Cologne Excellence Cluster on Cellular Stress Responses in Aging-Associated Diseases (CECAD), University of Cologne, Joseph-Stelzmann-Strasse 52, 50931 Cologne, Germany; 2 Institutes of Biomedical Sciences, Shanxi University, 030006 Taiyuan, China

## Abstract

SUN1, a component of the LINC (Linker of Nucleoskeleton and Cytoskeleton) complex, functions in mammalian mRNA export through the NXF1-dependent pathway. It associates with mRNP complexes by direct interaction with NXF1. It also binds to the NPC through association with the nuclear pore component Nup153, which is involved in mRNA export. The SUN1-NXF1 association is at least partly regulated by a protein kinase C (PKC) which phosphorylates serine 113 (S113) in the N-terminal domain leading to reduced interaction. The phosphorylation appears to be important for the SUN1 function in nuclear mRNA export since GFP-SUN1 carrying a S113A mutation was less efficient in restoring mRNA export after SUN1 knockdown as compared to the wild type protein. By contrast, GFP-SUN1-S113D resembling the phosphorylated state allowed very efficient export of poly(A)+RNA. Furthermore, probing a possible role of the LINC complex component Nesprin-2 in this process we observed impaired mRNA export in Nesprin-2 knockdown cells. This effect might be independent of SUN1 as expression of a GFP tagged SUN-domain deficient SUN1, which no longer can interact with Nesprin-2, did not affect mRNA export.

## Introduction

SUN1 is an inner nuclear membrane protein and is a member of the SUN domain containing proteins that are composed of a variable N-terminus facing the nucleoplasm, a transmembrane domain and the C-terminal SUN domain through which they can interact with the KASH domain of KASH domain containing proteins in the perinuclear space. In mammals, five SUN paralogs have been described, the ubiquitous SUN1 and SUN2, and testis-specific SUN3, SUN4 and SUN5^1^. SUN proteins are central components of the LINC complex that connects the interior of the nucleus to the cytoskeleton^2, 3^. This complex has functions in nuclear migration, nuclear positioning, maintaining nuclear shape, mechanotransduction and chromosome movement in meiosis^4^. Sun1/Sun2 double knockout in mice is neonatal lethal which might be indicative of important functions in development^5^.

In our previous work we focused on a role of SUN1 in relation to the nuclear pore complex (NPC) as an association with the NPC had been reported^6, 7^. In particular, we found that SUN1 is involved in NXF1 (nuclear export factor 1) dependent nuclear export of mRNAs through interactions with hnRNPs (heterogeneous nuclear ribonucleoproteins), NXF1 and NUP153^8^. Knockdown of SUN1 led to accumulation of mRNA in the nucleus that could be rescued by re-expression of SUN1. We proposed that mRNPs in complex with NXF1 attach to SUN1 at the nuclear envelope (NE). The mRNP/NXF1 complexes are then passed on to NUP153, which takes them to the nuclear pore and guides them through the NPC. *In vitro* binding studies using recombinant proteins revealed a direct interaction of the N-terminus of SUN1 with NXF1:NXT1 and NUP153. NXF1 and NXT1 form a heterodimer and function together in mRNA export^9^. For NUP153, the interaction with SUN1 occurred in its N-terminal domain and in the FG-repeat containing C-terminal domain^8^. It is unclear whether and how the interaction between the various components is regulated. We investigated here the possibility that phosphorylation is a means of regulation.

The NPC-dependent mRNA export is divided into two main pathways, the CRM1-dependent and the NXF1-dependent pathway. The CRM1-dependent pathway is used by rRNAs, snRNAs and a subset of mRNAs, whereas the NXF1-dependent pathway is responsible for export of most mRNAs. For export, mRNAs need to be properly processed; they associate with proteins such as hnRNPs and SR proteins to form mRNPs which then bind to the TREX (transcription-export) complex and finally interact with the export receptor NXF1:NXT1. mRNP/NXF1 recognize nucleoporins with FG-domains at the NPC which mediate docking at the nucleoplasmic side and translocation through the NPC transport channel and finally release into the cytoplasm^10, 11^.

SUN1 phosphorylation has been reported previously. This phosphorylation is occurring during mitosis, is carried out by Cdk1 and Plk1 and leads to a weaker interaction of SUN1 with LaminA, Emerin and Nesprins. The three phosphorylation sites are located in the N-terminal part^12^. Furthermore, SUN1 has been identified as interaction partner of DNA-dependent protein kinase catalytic subunit (DNA-PKcs) in a biochemical screen, but phosphorylation studies were not carried out^13^. SUN1 and SUN2 phosphopeptides were also found in proteome analyses of skin^14^. We identified potential phosphorylation sites for protein kinase C (PKC) in the N-terminus of SUN1 and investigated the role of this phosphorylation in SUN1 interactions with different binding partners and in mRNA export. PKCs are a family of Serine/Threonine kinases with regulatory roles in many cellular processes. They are present in the cytoplasm, however, nuclear localization and nuclear roles have also been shown^15^. Furthermore, based on reports that GANP (germinal center-associated nuclear protein) promotes export of a subset of mRNAs, we investigated whether particular mRNAs use the export pathway involving SUN1^16^. GANP is a subunit of the TREX-2 mRNA export complex and interacts with NXF1^17^. We found this protein also in complex with SUN1^8^. In addition, we studied whether the LINC complex is involved in mRNA export as well and whether it cooperates with SUN1.

## Results

### Regulation of SUN1 in nuclear mRNA export

**Figure 1:**
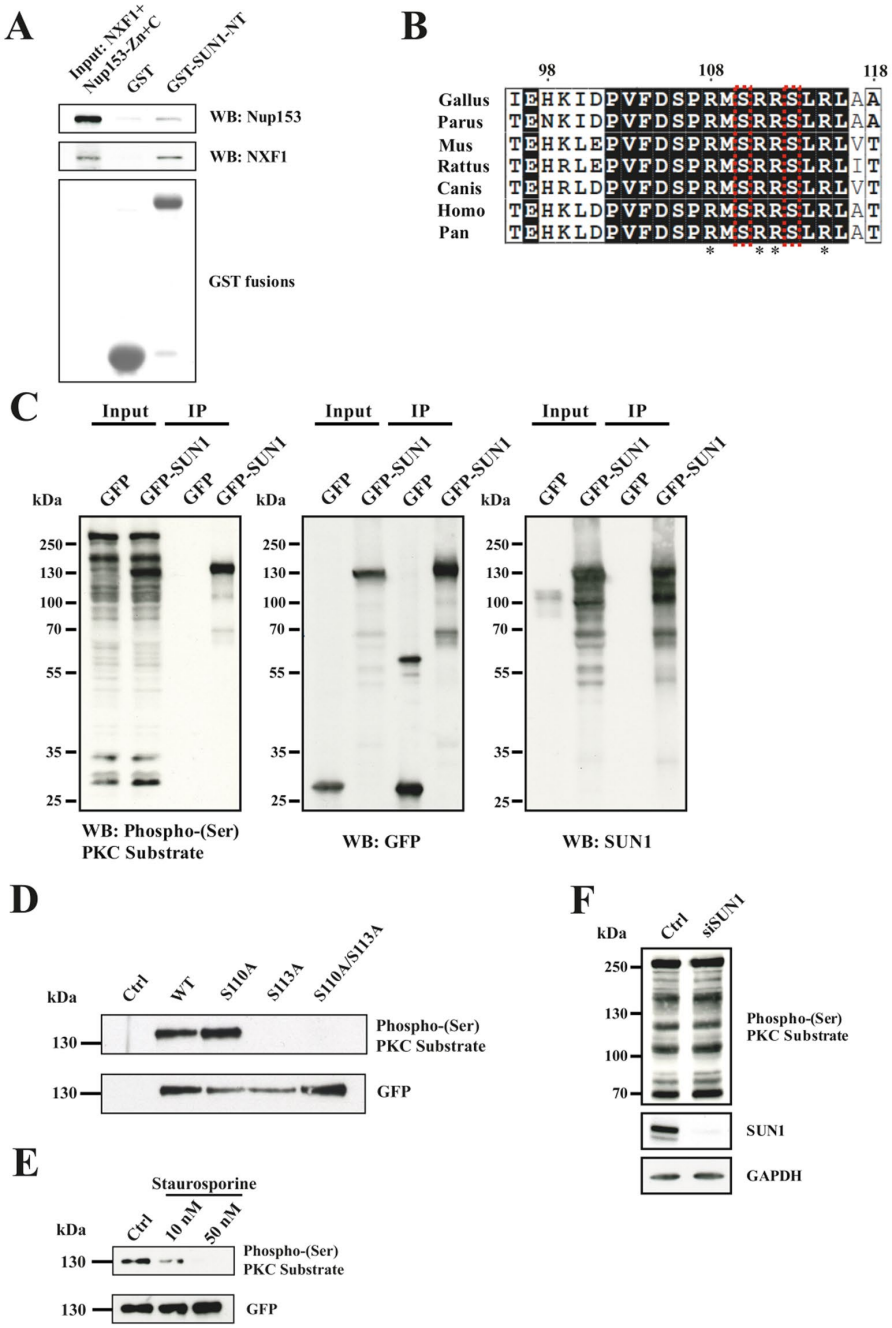
SUN1 interacts differentially with NUP153 and NXF1 and is a substrate of protein kinase C. (**A**) Competition between NXF1 and NUP153 for SUN1 binding. Recombinant NXF1/NXT and Nup153-Zn+C released from GST (input) were used for pull down experiments with GST (control) and GST-SUN1-NT. NXF1 and NUP153 were detected by antibodies. Below the pull down a western blot detecting the GST proteins is shown. (**B**) Comparison of the highly conserved predicted PKC phosphorylation site in SUN proteins from various species. The numbering corresponds to human SUN1 (ACCESSION: EAL23707). The S residues S110 and S113 are boxed in red. Stars point out the consensus sequence. Boxed in black, identical amino acid residues, boxed in white, similar amino acid residues. (**C**) Cell lysates from GFP and GFP-SUN1 expressing HeLa cells (Input) were used for pull downs using GFP trap beads (IP) and probed with Phospho-(Ser) PKC Substrate, GFP and SUN1 antibodies as indicated. (**D**) WT SUN1 and SUN1 proteins carrying point mutations at the indicated positions were expressed as GFP fusions in HeLa cells and precipitated using GFP trap beads. The blot was probed with Phospho-(Ser) PKC Substrate and GFP antibodies. For control, cells expressing GFP were used. (**E**) Staurosporine treatment reduces SUN1 phosphorylation. Staurosporine was applied for 6 hours at the indicated concentrations. The immunoprecipitated material was probed with Phospho-(Ser) PKC Substrate followed by GFP antibodies. (**F**) SUN1 knockdown does not affect the staining pattern of Phospho-(Ser) PKC Substrate antibodies in whole cell lysates. siRNA mediated SUN1 knockdown was confirmed by labelling with SUN1 antibodies. GAPDH was used as loading control.

NXF1:NXT1 and NUP153 act together with SUN1 in nuclear mRNA export^8^. How these interactions are regulated in order to guide and allow the export process is unknown. Potential mechanisms are differential affinities of the components involved or regulations through modifications of the proteins such as phosphorylation. We therefore asked first whether SUN1 has different preferences for NXF1:NXT1 and NUP153 by performing a qualitative assay in which we compared the amounts of recombinant NXF1 and NUP153 in the input with the ones in the pulldown using GST-SUN1-NT (residues 1–239) bound to beads. We found that NXF1 was more abundant in the pull down as compared to NUP153 despite the comparatively stronger NUP153 signal in the input (Fig. 1A). This suggests a difference in the affinities of SUN1 for both proteins which might play a role in mRNA export. The GST control did not bind the proteins. A further mechanism could be a regulation of the interaction by phosphorylation. SUN proteins are phosphorylated by several kinases during mitosis and have also been identified as interaction partners of DNA-PK^12, 13^. We used the Netphos3.1 prediction program (www.cbs.dtu.dk/services/NetPhos/) to search for potential phosphorylation sites and found two overlapping copies of the PKC recognition motif R/KXpSXR/K in the N-terminus of SUN1 at position S110 and S113. This region is highly conserved among SUN1 proteins from different species (Fig. 1B).

For the PKC recognition motif a Phospho-(Ser) PKC Substrate antibody is available which recognizes proteins phosphorylated by PKC. To analyze whether these sites are in fact substrates of a PKC, we expressed GFP-SUN1 and GFP for control in HeLa cells and precipitated the proteins using GFP-Trap beads. The resulting blot was probed with the Phospho-(Ser) PKC Substrate antibody, which in the whole cell lysate labelled many proteins. Prominent bands were present above 250 kDa and above 130 kDa in GFP and GFP-SUN1 expressing cells. In the lysate from GFP-SUN1 transfected cells an additional strong band was present at ~130 kDa. In the GFP-SUN1 pull-down a band of ~130 kDa was recognized by the Phospho-(Ser) PKC Substrate antibody. In the control pull-down from cells expressing GFP no signal was present. After stripping of the membrane and reprobing with GFP specific mAb K3–184–2 followed by reprobing with SUN1 antibodies, the ~130 kDa protein was identified as GFP-SUN1 showing that SUN1 indeed is a PKC substrate (Fig. 1C). We then performed similar assays with GFP-SUN1 proteins carrying the non-phosphorylatable S110A, S113A and S110A/S113A mutations and found that the S113A and S110A/S113A double mutant were no longer recognized by the Phospho-(Ser) PKC Substrate antibody in contrast to the single S110A mutant. These results identify S113 as the PKC phosphorylation site. GFP antibodies demonstrated successful precipitation of the protein (Fig. 1D). To obtain independent support for PKC involvement in SUN1 phosphorylation we used Staurosporine, a general inhibitor of PKCs. We treated HeLa cells expressing GFP-SUN1 with Staurosporine (10 and 50 nM) for six hours, precipitated the GFP-tagged protein and probed the precipitate with the Phospho-(Ser) PKC Substrate antibody. The 130 kDa protein was detected in the untreated control, a faint signal was seen in the precipitate from cells treated with 10 nM Staurosporine, and no signal was present in the precipitate from cells treated with 50 nM Staurosporine. Reprobing the blot with GFP-specific antibodies showed the presence of GFP-SUN1 in all three samples confirming the phosphorylation by a PKC (Fig. 1E). The concentrations of Staurosporine used did not impair the growth of the cells. We also tested whether knockdown of SUN1 in HeLa cells by siRNA treatment affected the overall phosphorylation pattern but did not observe changes indicating that SUN1 is not an abundant substrate (Fig. 1F).

**Figure 2:**
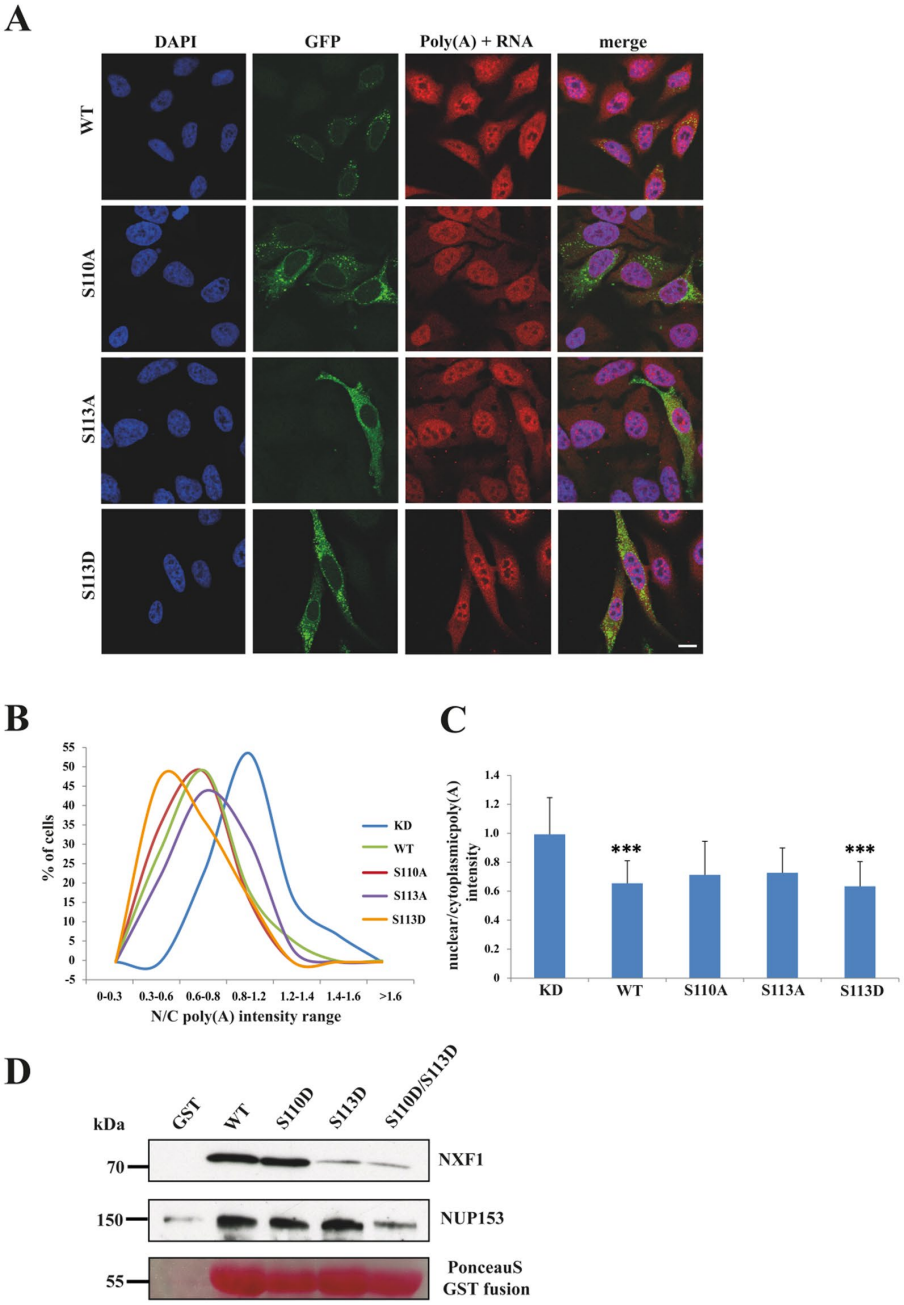
Impact of serine 110 or 113 point mutations in the PKC recognition motif on mRNA export. (**A**) RNA FISH analysis shows differential distribution of nuclear and cytoplasmic RNA in HeLa cells treated with siRNA specific for SUN1 and transfected with GFP-SUN1^R^ expression vectors carrying the indicated mutations. Cells not expressing GFP fusion proteins represent SUN1 depleted cells. Size bar, 10 µm. (**B**) The nuclear/cytoplasmic (N/C) ratio of the poly(A)+RNA distribution in the various strains as determined by measuring the fluorescence intensity. The percentages of cells in the indicated N/C intensity ranges are shown. The results from one typical experiment are shown. The graph on the right shows the mean values with standard deviations (P value, ***< 0.001). (**C**) The graph on the right shows the mean values with standard deviations (P value, ***<0.001). (**D**) Effect of the S to D mutations in GST-SUN1-NT on the interaction with NXF1 and NUP153. GST and GST fusion proteins were used to pull down NXF1 and Nup153 from HeLa cell lysates. The PonceauS stained gel part shows the GST fusion proteins.

To probe whether phosphorylation of the SUN1 N-terminus has an impact on mRNA export we used GFP-SUN1 proteins carrying S110A and S113A mutations for rescue experiments in SUN1 depleted cells. FISH analysis was carried out for GFP positive cells and the nuclear/cytoplasmic (N/C) ratio determined. The results showed that in GFP-SUN1-WT and GFP-SUN1-S110A expressing cells the poly(A)+RNA was enriched in the cytoplasm with N/C ratios peaking in the range of 0.6–0.8, whereas in GFP-SUN1-S113A expressing cells an accumulation in the nucleus was observed with the N/C ratio shifted towards the right showing a broad peak. This reflected higher nuclear accumulation and, since knockdown cells had a N/C ratio centered in the range of 0.8–1.2, less efficient rescue of the knockdown phenotype. We then generated GFP-SUN1-S113D resembling the phosphorylated form and found a strong accumulation in the cytoplasm and an N/C ratio peaking in the range of 0.3–0.6 (Fig. 2A–C). Phosphorylation of S113 therefore appears to be a regulatory factor and responsible for efficient SUN1 mediated mRNA export.

We next mutated S110, S113 and S110/S113 to D residues in GST-SUN1-NT in order to study whether the interaction of NXF1 and Nup153 with SUN1 is affected by phosphorylation. The fusion proteins were used to precipitate NXF1 and NUP153 from HeLa cells. In these experiments we found that GST-SUN1-NT-S113D and GST-SUN1-NT-S110D/S113D consistently precipitated lower amounts of NXF1 than GST-SUN1-NT-WT and GST-SUN1-NT-110D. However, the interaction was not completely abrogated, which appears relevant for initial binding of the mRNP/NXF1 complex and indicates that additional factors influence binding and release. NUP153 did not appear to be affected (Fig. 2D). We conclude that phosphorylation could provide a mechanism with which the mRNA complex is released from SUN1 and becomes available for association with NUP153 and export through the nuclear pore complex.

### Is SUN1-mediated nuclear mRNA export selective?

**Figure 3:**
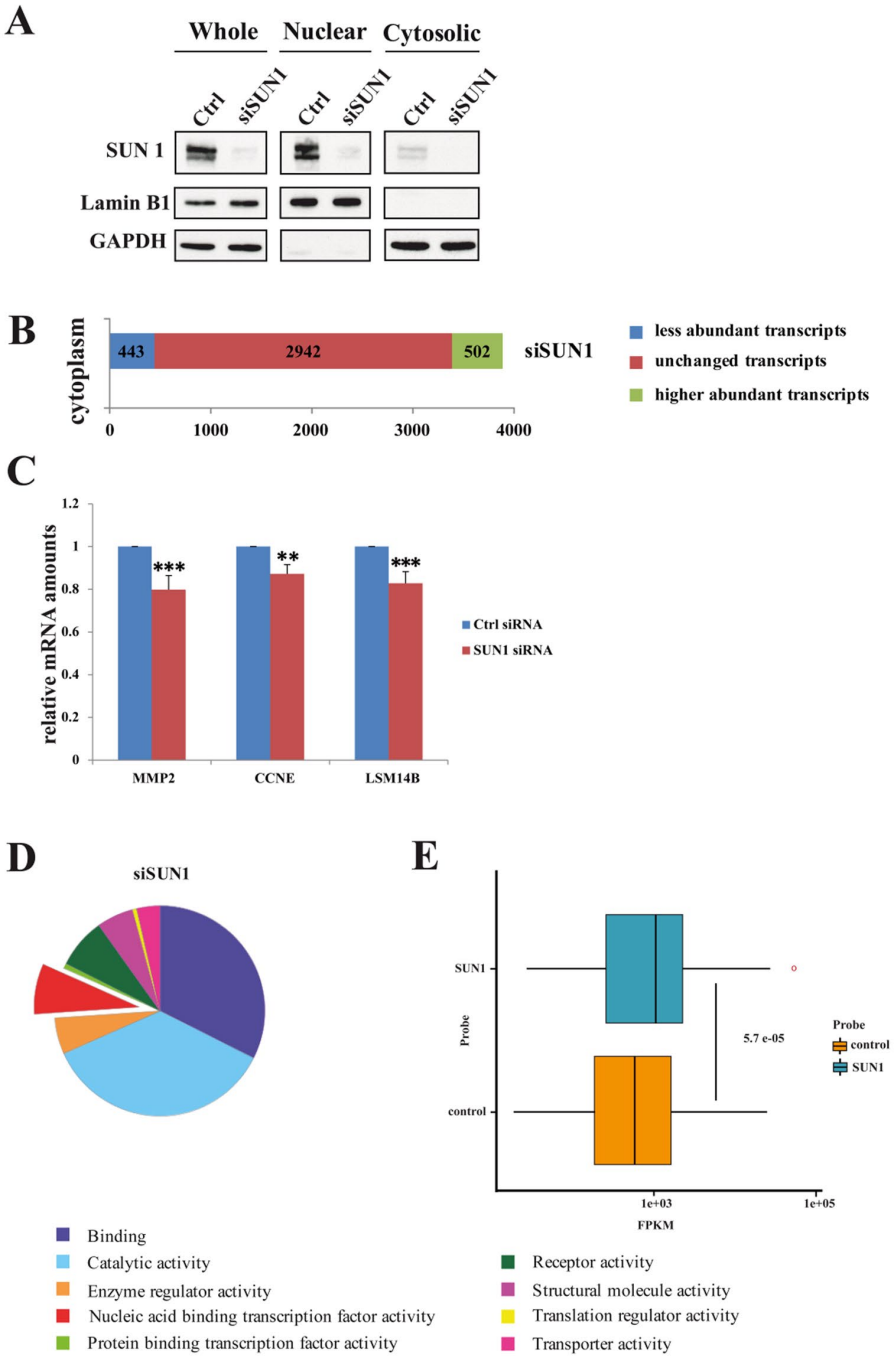
Whole genome expression profile of HeLa cells depleted of SUN1. (**A**) Fractionation of control and SUN1 knockdown cells into nuclear and cytosolic fraction. LaminB is a marker for the nucleus, GAPDH a marker for the cytosol. (**B**) The total number of less abundant, unchanged and higher abundant transcripts in the cytoplasm compared to control knockdown (Ctrl) identified in the analysis is shown. The data result from three independent knockdown experiments. (**C**) Validation of the results for three transcripts by q-RT PCR. MMP-2, matrix metalloproteinase-2; CCNE, cyclin E1; LSM14B, protein LSM14 homolog B. P value **<0.01, ***<0.001. (**D**) GO molecular function categorization of the mRNAs depleted in the cytoplasm of cells with reduced SUN1 levels. (**E**) Genes, whose transcripts are less common in the cytoplasm of SUN1 knockdown than in the control are more highly expressed in the control than the average gene. SUN2 knockdown was used for comparison (control). The data represent the results of three individual knockdowns. FPKM = Fragments Per Kilobase of exon per Million reads mapped. The graphics was produced using ggplot implemented in R. The Wilcoxon signed-rank test was used to test for the significance of the results.

Wickramasinghe *et al*.^16^ reported that GANP and NXF1, which are both components of the TREX-2 mRNA export complex, promote export of specific mRNAs. These transcripts are enriched for those that encode proteins involved in gene expression and have roles in RNA biology such as processing and mRNP biogenesis. Since SUN1 interacts with GANP and NXF1 it might also play a role in selective mRNA export^8^. We therefore performed sequencing of RNA isolated from the cytoplasm of SUN1 and control knock down cells (Fig. 3A). In SUN1 depleted cells 2942 cytoplasmic transcript levels were unchanged as compared to the control siRNA treated cells. 443 transcripts were less abundant in the cytoplasm (blue in Fig. 3B) after SUN1 depletion and 502 transcripts (green in Fig. 3B) were found to be more abundant. For three randomly chosen transcripts we confirmed the reduction by qRT-PCR (Fig. 3C).

To classify the encoded protein products of the differentially regulated genes, they were loaded onto the KEGG Automatic Annotation Server (http://www.genome.jp/tools/kaas/). This way we annotated the genes with corresponding KEGG IDs. A categorization of the mRNAs depleted from the cytoplasm after SUN1 protein knock down using GO molecular function analysis did not show an enrichment of genes belonging to specific classes suggesting that SUN1 has a general effect on mRNA export (Fig. 3D). We then took the expression levels of the genes into account and compared the cytosolic mRNAs from SUN1 knockdown cells with those from SUN2 knockdown cells (control). SUN2 knockdown was taken as wild type control since it did not impair mRNA export (data not shown). We observed that genes, whose products were less common in the cytoplasm of the SUN1 mutant as compared to the wild type control, tended to be more highly expressed under normal conditions than the average gene (Fig. 3E). Thus, the SUN1 protein seems to have an additive role in the transport of more highly expressed genes from the nucleus into the cytosol.

### The LINC complex and mRNA export

**Figure 4:**
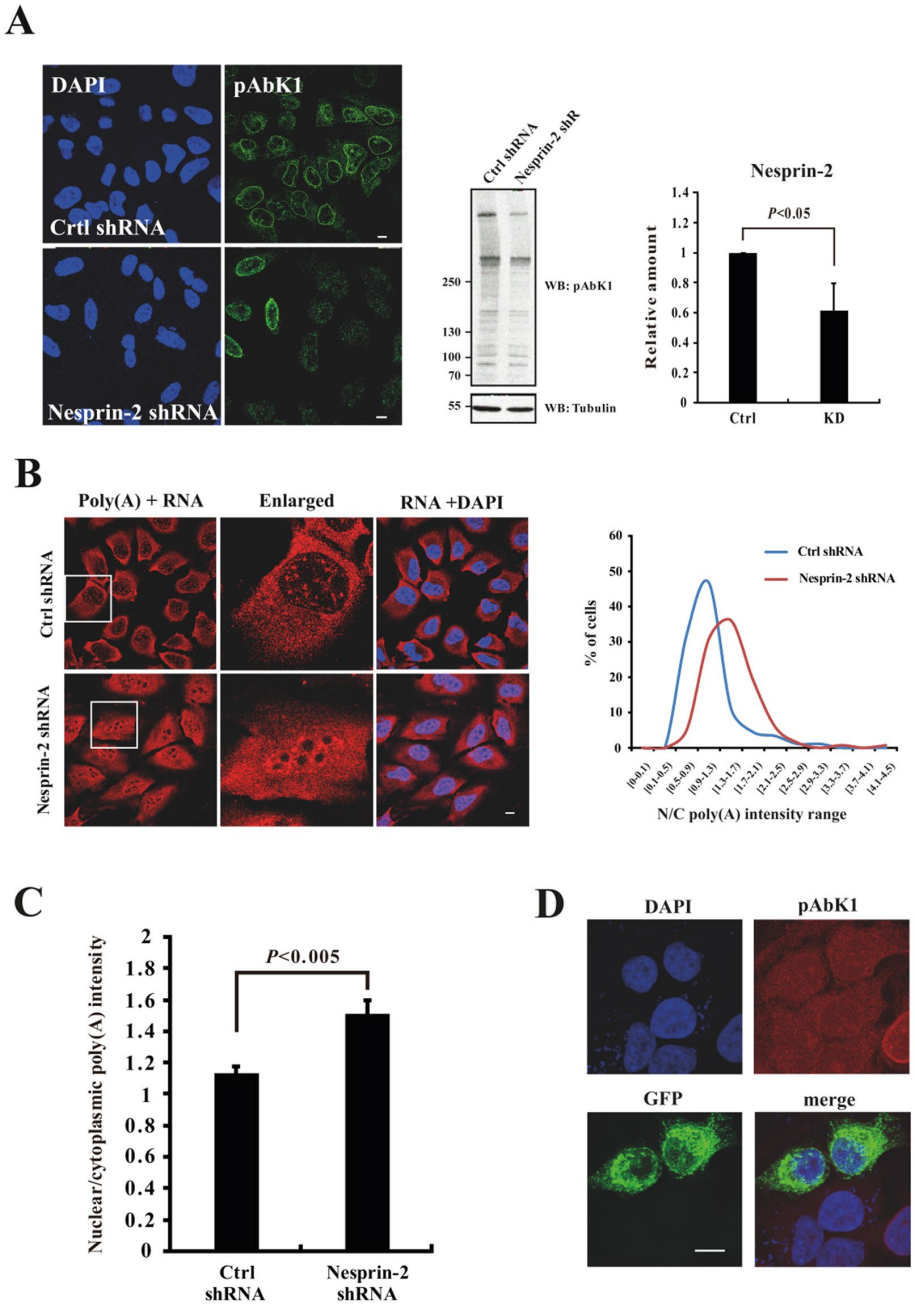
The LINC complex in mRNA export. (**A**) Efficiency of Nesprin-2 knockdown (KD) by shRNA treatment in HeLa cells is shown by immunofluorescence with Nesprin-2 specific antibodies pAbK1 and by western blot analysis. The quantitative determination of the knockdown is depicted in a bar graph (left). Scale bar, 5 µm. (**B**) RNA FISH analysis of Nesprin-2 depleted cells as shown by immunofluorescence analysis. Left side, distribution of the N/C intensity range. (**C**) The mean nuclear/cytoplasmic (N/C) intensity ratio of control and Nesprin-2 KD cells. The values represent the mean ± SD of more than 100 cells analyzed. (**D**) Immunofluorescence analysis of GFP-SUN1-ΔSUN expressing cells. Nesprin-2 was detected by pAbK1. Scale bar, 10 µm.

SUN proteins form a complex with Nesprins through interaction of their SUN domain with the KASH domain of Nesprins in the perinuclear space^2–4^. This complex has many roles and also allows a crosstalk between the cytoplasm and the nucleus^4^. In analogy to a proposal made by Chang *et al*.^4^, Nesprins could help anchoring SUN1 near the NPC to enable it to fulfill its task in mRNA export. Interestingly, in our initial studies of Nesprin-2 (NUANCE) we reported partial colocalization of Nesprin-2 with NUP358^18^. Here we studied the mRNA export in Nesprin-2 depleted cells by FISH analysis. The depletion was achieved by plasmid based shRNAs targeting the N- and C-terminus of Nesprin-2^19, 20^. Confirmation was by western blot and immunofluorescence analysis (Fig. 4A). In cells treated with control shRNA the majority of the cells showed a N/C ratio with a peak at ~0.9. In Nesprin-2 depleted cells the N/C ratio was shifted to the right peaking at ~1.3 indicating a significantly impaired mRNA export (Fig. 4B,C). This reduction could be due to an effect of Nesprin-2 based on its association with the NPC or due to the disturbance of the LINC complex. To distinguish between these possibilities, we expressed GFP-SUN1-ΔSUN in HeLa cells and performed a FISH analysis. GFP-SUN1-ΔSUN is still anchored in the inner nuclear membrane, however it can no longer bind to Nesprins due to the absence of the SUN domain. This leads to the loss of Nesprin-2 from the NE (Fig. 4D). The FISH analysis showed that in GFP positive cells poly(A) +RNA export was not much different from HeLa cells (mean N/C ratios of 0.81 for GFP-SUN1-ΔSUN positive cells and 0.88 for HeLa cells).

## Discussion

**Figure 5:**
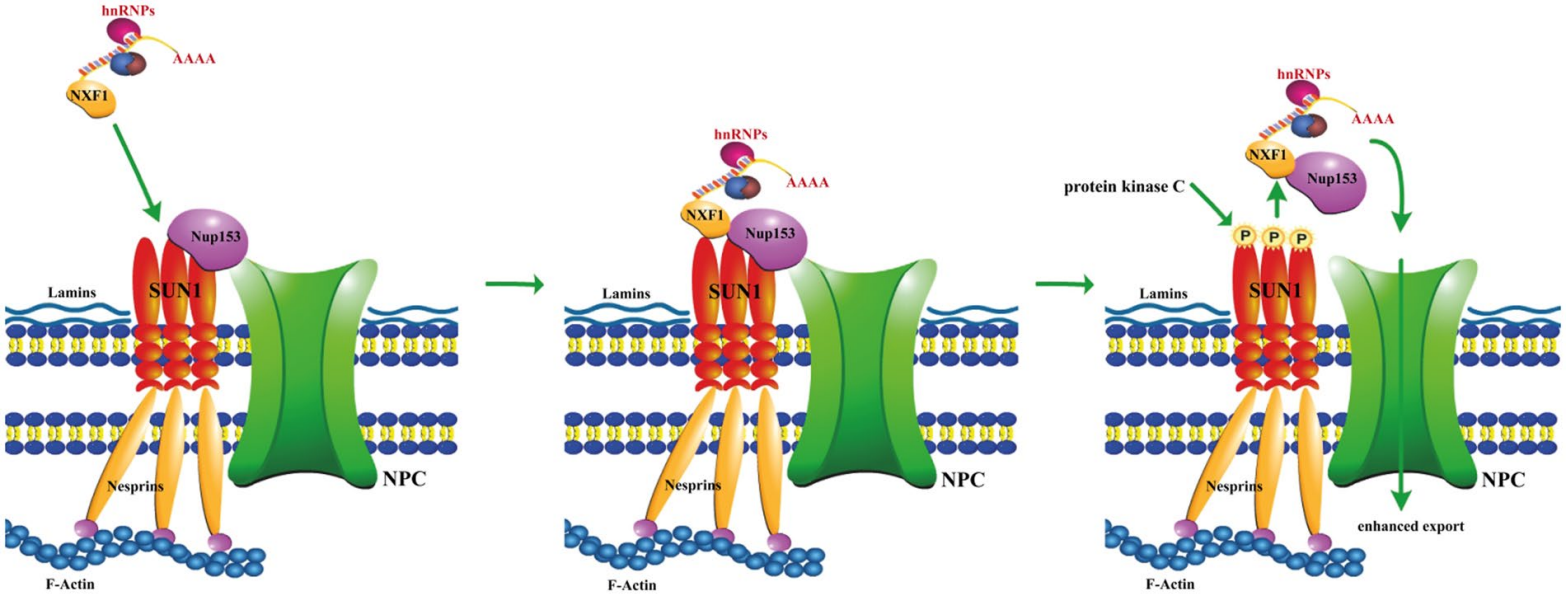
Model depicting the proposed role of SUN1 phosphorylation in mRNA export. SUN1 binds mRNP/NXF1 complexes at the nuclear envelope (NE). Upon an unidentified signal, SUN1 is phosphorylated at S113 by a PKC. This reduces the NXF1 affinity and the complex then binds to NUP153 which is also associated with SUN1 at the NE. The mRNP is targeted to the nuclear pore and exported.

The NXF1:NXT1 complex and NUP153 interact with the amino terminus of SUN1. The binding sites in NUP153 are located in the N- and the C-terminal region. The C-terminal region of NUP153, which is the FG rich domain, carries also the interaction site for NXF1^21^ and there could be some competition between the three proteins through differential binding. Our *in vitro* binding studies suggest that NXF1:NXT1 has a higher affinity to SUN1 than NUP153. Phosphorylation of SUN1 at S113 could reduce the NXF1 binding and allow transfer of the mRNP/NXF1 complex to NUP153 (Fig. 5). Accordingly, mutation of this residue to alanine in GFP-SUN1 led to a molecule that was less effective in rescuing the mRNA export in SUN1 knock down cells as we observed elevated accumulation of mRNA in the nucleus in FISH experiments. By contrast, GFP-SUN1-S113D mimicking the phosphorylated state behaved similar to GFP-SUN1. In fact, mRNA export was more efficient. GFP-SUN1-S113D resembles a permanently phosphorylated molecule and its presence could lead to strongly increased mRNA export. This appears to be not the case since we observed only moderately higher export as compared to the wild type protein. There could be several reasons; GFP SUN1-S113D could form hetero-oligomers with residual endogenous SUN1 or phosphorylation is only one regulatory mechanism among others. This latter proposal is supported by the pull down results which show that the S113D mutation does not completely abolish the interaction between SUN1 and NXF1:NXT1. It could also be that both proposals come into play.

S113 is contained within a predicted PKC phosphorylation site. By expressing wild type and mutant GFP-SUN1 and using an antibody recognizing the phosphorylated site in combination with results from Staurosporine treatment we could confirm the PKC phosphorylation site. Several PKC isoforms exist and they have regulatory roles in many cellular processes. In general, they are cytoplasmic enzymes that translocate to the plasma membrane upon activation by lipids. However, nuclear translocation has also been reported for several PKC isozymes, and a number of nuclear substrates reaching from histones, lamins, hnRNPs to RNA-Polymerase II has been identified^15^. For PKCε a localization to the NPC was reported and therefore it could be the kinase phosphorylating SUN1^22^. As PKCs are regulated by lipid messengers this could be a mechanism by which export of mRNA could be enhanced. PKCs are important regulators of cell proliferation and differentiation^15^. Phosphorylation of SUN1 might help to export mRNAs to the cytoplasm for these processes. The findings from mRNA sequencing that the products of more highly expressed genes are affected by SUN1 knockdown also supports such a role. Thus, through our investigations we have found a further potential nuclear substrate of PKC and attributed a role to the PKC phosphorylation of SUN1.

When we tested the possibility whether SUN1 is involved in the export of specific mRNAs we did not obtain any indication for such a role. We did however observe that the affected mRNAs that were less common in the SUN1 mutant cytoplasm than in the control WT tended to be derived from genes that are more highly expressed in the control than the average gene. The basis of such a distinction is not known. For the GANP/NXF1 mediated export it was found that it is playing a role in export of mRNAs which are highly expressed, have short half-lives and code for proteins that allow rapid adaptation to changes in gene expression, whereas transcripts that rely on NXF1 have long half-lives and code for proteins required for cellular metabolism^16^. The number of transcripts identified in this study was significantly higher than in our work and more than 10.000 transcripts were detected. Whether this is due to an influence of SUN1 depletion on transcription in general is not known. In our previous work we tested for cell viability in knock down cells and observed a reduction after 72 hours of depletion^8^. Also, we relied in our studies on RNA-Seq for mRNA analysis, whereas the study by Wickramasinghe used Expression Bead Chips (Illumina) for hybridization^16^.

SUN1 is an essential component of the LINC complex and interacts through its SUN domain with the C-terminus of Nesprins in the periplasmic space that is formed by the two nuclear membranes^23^. Based on the existence of this complex it could well be that Nesprin is also involved in mRNA export. We chose Nesprin-2 for our analysis since we had previously shown that it colocalizes to some extent with nuclear pore complexes^18^. Knockdown of Nesprin-2 reduced mRNA export, whereas disturbance of the Nesprin SUN interaction by expressing a SUN domain-less SUN1 protein did not affect export and implies a SUN1 independent action of Nesprin-2. However, since Nesprin-2 interacts with components of the cytoskeleton, the possibility exists that mRNA export is coupled to signals or forces coming from the cytoplasm and the extracellular space as has been suggested by Jahed *et al*. recently even if this does not involve the LINC complex in combination with SUN1^23^.

## Materials and Methods

### Cell culture and drug treatment

HeLa cells were cultured in Dulbecco’s modified Eagle’s medium supplemented with 10% fetal bovine serum, penicillin and streptomycin (100 μg/ml) and L-glutamine at 37 °C with 5% CO_2_. Staurosporine (Sigma) was used as general protein kinase C (PKC) inhibitor. HeLa cells were treated with 10 and 50 nM Staurosporine for six hours and then used for the analysis. Under these conditions growth was not impaired.

### Plasmids, site-directed mutagenesis, small interfering RNA-(siRNA)-mediated depletion and shRNA mediated knockdown

Plasmids encoding human full length SUN1 (GFP-SUN1) and the siRNA resistant (R) GFP-SUN1^R^ as well as GST-SUN1-NT (residues 1-239) have been described previously (SUN1 Accession number O94901)^8^. Mutations to generate GFP-SUN1 or GFP-SUN1^R^ S110A, S113A, S110A/113A and S113D and GST-SUN1-NT S110D, S113D, S110D/S113D were introduced using a site-directed mutagenesis kit (Promega). GFP-SUN1^R^-ΔC contains residues 1 to 412 encompassing the N-terminus and the transmembrane region but lacking the coiled coil region and the SUN domain. Transfections of HeLa cells were carried out using Lipofectamine 2000 (Invitrogen) or o-fekt (Bio-Budget Technologies, Krefeld, Germany). Analyses were done 24 to 48 hours after transfection. For siRNA-mediated depletion, HeLa cells were transfected with SUN1 targeted siRNA or control siRNA described previously^8^ and SUN2 targeted siRNA (GE Healthcare Dharmacon). For RNAi transfection, the reagent INTERFERin (Polyplus) was used. Cells were analyzed 72 to 96 hours after transfection. For rescue experiments, cells were transfected with GFP-SUN1^R^ or GFP-SUN1^R^-ΔC plasmids using Lipofectamine 24 h after siRNA treatment. Analyses were done 72 to 96 hours after siRNA treatment^8^.

sh RNA knockdown of Nesprin-2 was carried out using oligonucleotides targeting N- and C-terminal sequences of Nesprin-2 Giant as described^19, 20^.

### RNA FISH

RNA fluorescence *in situ* hybridization (FISH) was done as described^8^. 5 × 10^3^ cells were seeded per well (24 well plate) and fixed at 72 to 96 hours after RNAi transfection. Fixation was with 4% PFA for 15 min. Then the cells were permeabilized by 0.2% Triton X-100 in PBS (phosphate buffered saline) for 5 min at room temperature (RT). Between each step, the cells were washed 3 times (5 min each) with PBS. Pre-hybridization was performed by incubating the cells with prehybridization buffer at 37 °C for 1 hour in a tissue culture incubator. The poly(A)+RNA was localized by hybridization with diluted Cy3-conjugated Oligo d(T)50 probe (Gene Link, Inc) (1 ng/μl) in prehybridization buffer at 37 °C for 2 hours in a tissue culture incubator. Prehybridization buffer was freshly prepared before use (for 1 ml: 2.5 µl tRNA (50 mg/ml), 50 µl herring sperm DNA (10 mg/ml), 100 µl SSC (20x), 50 µl BSA (20 mg/ml), 500 µl formamide, 5 µl vanadyl ribonucleoside complexes (200 mM), 0.1 g dextran sulfate, 289.5 µl ddH_2_O). Hybridization was followed by several washing steps. Washing was for 3 times (10 min each) in 2xSSC at 37 °C, 3 times (10 min each) in 1xSSC at RT and 3 times (5 min each) in PBS. DNA was stained with DAPI diluted in 0.2% Triton X-100/PBS for 30 min at RT. The cells were washed in PBS and mounted with gelvatol. Images were acquired by confocal laser scanning microscopy (TCS-SP5, Leica). Processing was with LAS AF Lite software (Leica). The mean Cy3 intensity was determined for the cell and the nucleus. The cytoplasmic pixel intensity was calculated and the nuclear/cytoplasmic (N/C) ratio determined. Three to four independent experiments were performed and 30 to 40 cells were analyzed per experiment or as indicated. Statistical analysis was done using Exel and Graph Pad software.

### Antibodies

Rabbit polyclonal anti-SUN1 antibodies (ab103021) were from Abcam, rabbit polyclonal Phospho-(Ser) PKC Substrate Antibody (#2261) was from Cell signaling. GFP was detected by mAb K3-184-2^21^. For Nesprin-2, mAb K20-478 and pAbK1 were used^18, 19^. Mouse monoclonal anti-NXF1, mouse monoclonal anti-Nup153 and rabbit anti-LaminB1 polyclonal antibodies were from Abcam, mouse monoclonal anti-GAPDH-POD used in western blots was from Sigma. mAb K3-184-2 was used to detect GFP in western blots^24^. Membranes were stripped of antibodies by incubation with 0.1 M NaOH for 5 min for two times and reused for immunolabelling. The antibodies were used at concentrations recommended by the supplier.

### RNA isolation, RNAseq and quantitative real time PCR (qRT-PCR)

For isolation of cytoplasmic RNA from control and siRNA treated cells, cells were fractionated into cytosolic and nuclear fractions. For this, cells were lysed by addition of 0.1% NP40 in PBS, pH 7.4, nuclei were pelleted and the supernatant used for cytoplasmic RNA isolation using RNeasy Mini Kit (Qiagen) according to the manufacturer’s protocol. Separation of nuclear and cytoplasmic fractions was assessed by analyzing cytosolic and nuclear fraction for the presence or absence of GAPDH and LaminB1 as marker by SDS-PAGE, respectively. The knock down was confirmed by probing with SUN1 specific antibodies. The quality of the total RNA was evaluated using both agarose gel electrophoresis and Bioanalyzer 2100 (Agilent), the concentration was assessed via NanoDrop ND-8000 spectrophotometry (Thermo Scientific). RNA was prepared from three independent experiments.

For mRNA-Seq sample preparation, the TruSeq stranded mRNA library prep kit (Illumina) was used. 1 µg of each total RNA sample was used for polyA mRNA selection using streptavidin-coated magnetic beads. The polyA selected mRNA was fragmented and amplified for cDNA synthesis using reverse transcriptase and random hexamer priming. The amplified cDNA was then converted into double stranded cDNA, end repaired and adaptors were added by ligation. Size selection was performed using gel purification (2% agarose gel). The generated cDNA libraries ranging in size from 200–250 bp were amplified by PCR (15 cycles) and quantified using the Bioanalyzer 2100 (Agilent). Each library was run at a concentration of 7 pmol using paired-end 75 bp sequencing on Hiseq. 4000 device (Illumina). The sequencing reads were aligned to the hg19/GRC37 reference genome (Ensembl 75). For comparison, cytoplasmic mRNA from cells in which SUN2 was depleted using siRNA was used. Differences in abundance of mRNAs were assessed by mapping the reads to the genome sequence and significance of differences was tested using the R package DEseq^25^.

### Pull down and immunoprecipitation experiments

Pull downs and immunoprecipitations were done as described^8^. Expression of GST-NXF1:NXT1 polypeptide was in *E. coli* XL1-Blue. GST-SUN1-NT and all GST-Nup153 polypeptides were produced in *E. coli* ArcticExpress RIL^8^. *E. coli* cells were lysed in lysis buffer (50 mM Tris/HCl, pH 8.0, 150 mM NaCl, protease inhibitors) by freeze thawing followed by sonification. The supernatant was incubated with Glutathione Sepharose 4B beads (GE-Healthcare) for three hours or overnight at 4 °C for isolation of the fusion proteins. The beads were pelleted, washed and incubated with HeLa cell lysates for 1.5 to 3 hours at 4 °C. GST bound beads were used as control. Beads coupled with protein complexes were washed 3 times (500 g, 4 °C, 1 min) with modified radio-immunoprecipitation (RIPA) lysis buffer (50 mM Tris/HCl, pH 7.5, 150 mM NaCl, 1% NP-40, 0.5% sodium deoxycholate, protease inhibitors) and heated in 5x SDS sample buffer (95 °C, 5 min). Samples were analyzed using SDS-PAGE (10 or 12% acrylamide) followed by western blotting. Lysis of HeLa cells was in modified RIPA lysis buffer. Cell suspensions were passed through a 0.45 µm needle for 10 times and incubated for 15 min on ice followed by 10 sec sonication. The lysates were cleared by centrifugation at 12,000 rpm for 10 min at 4 °C and the supernatants used.

For pulldown of GFP-tagged proteins from HeLa cell lysates protein A sepharose beads loaded with mAb K3-184-2 were used and the pull down performed as described above. The precipitated proteins were analyzed in western blots.

### Data Availability

The RNAseq data are available via https://www.ncbi.nlm.nih.gov/biosample/6651674 under the BioSample accession SAMN06651674.
